# Identification of prognosis value and immune microenvironment features of ceRNAs in NSCLC with distinct gene mutation

**DOI:** 10.18632/aging.204846

**Published:** 2023-06-26

**Authors:** Jiangtao You, Qingshi Wang, Longlong Cong, Rui Zhao, Wei Cui, Huan Chen

**Affiliations:** 1 Department of Thoracic Surgery, The First Affiliated Hospital of Xi’an Jiaotong University, Xi’an, Shanxi, China; 2 Department of Surgical Oncology, The First People’s Hospital of Xianyang City, Xianyang, Shanxi, China; 3 Department of Vascular Surgery, The First Affiliated Hospital of Xi’an Jiaotong University, Xi’an, Shanxi, China; 4 Department of Endocrinology and Second Department of Geriatrics, The First Affiliated Hospital of Xi’an Jiaotong University, Xi’an, Shanxi, China

**Keywords:** NSCLC, mutation, ceRNA, immune, prognosis

## Abstract

Non-small cell lung cancer (NSCLC), representing about 85% of all lung cancer (LC) cases, is by far the most common form of LC. High-throughput technology largely expands our ability to analyze the transcriptome data and a plethora of cancer-driving genes has been identified, paving the path to immune therapy, where the effects of cancer-causing mutations are countered with microenvironment complexity. Given that competing endogenous RNAs (ceRNAs) participate in diverse cellular processes by a broad array of mechanisms in cancer, we scrutinized the immune microenvironment and ceRNA signatures in mutation-specific NSCLC by integrating TCGA-NSCLC and NSCLS-associated GEO datasets. The results suggested that RASA1mutation clusters in LUSC had a better prognosis and immunity. Immune cell infiltration analysis indicated that the cluster with RASA1 mutation had a significantly high level of NK T cells and a low level of memory effector T cells. Further analysis of immune-related ceRNAs in LUSC showed that hsa-miR-23a was significantly associated with survival in RASA1-mutation samples, indicating that there may be specific ceRNAs in mutation-specific subgroups in NSCLC. In conclusion, this study verified the presence of complexity and diversity of NSCLC gene mutations and highlighted the intricate links between gene mutation and tumor environment features.

## INTRODUCTION

Lung cancer (LC) is one of the most common causes of cancer death worldwide. Approximately 85% of LC with histological subtypes are identified as non-small cell lung cancer (NSCLC), of which lung adenocarcinoma (LUAD) and lung squamous cell carcinoma (LUSC) are the most prevailing subtypes [1]. NSCLC is a genetically heterogeneous disease with unique combinations of somatic mutation profiles and there are significant differences in genetic characteristics with regard to mutation spectrum and mutation frequencies [2]. Given that the soaring of large driver mutations in cancer, the treatment of NSCLC therapy has entered a new revolutionized era of targeted therapy [3]. Based on the increasing knowledge of driver mutations in NSCLC, a group of drugs targeting the well-known driver genes such as KRAS, EGFR, ALK, ROS1, MET, RET, NTRK, and RAF, have been approved for targeted therapy, which facilitates personalized medicine [4, 5]. Increasing gene variants and genetic characteristics have conferred LC to various sub-genotypes [6]. However, dissecting the complicated mutation pattern of NSCLC is one of the biggest challenges.

Competing endogenous RNAs (ceRNAs) are non-coding transcripts that can interact with each other at the post-transcription level by competing with the shared miRNAs [7]. CeRNAs contain microRNAs, long non-coding RNAs (lncRNAs), pseudogenic RNA, and circular RNA. Increasing evidence has revealed that ceRNAs play a crucial role in tumor immune infiltration, thus affecting the response to immune therapy [8, 9]. For example, LINC00973 expression launched by EGFR/Wnt signaling perceives the miR-216b/CD55 and miR-150/CD59 complex by ceRNA mechanism, which impedes the complement system activity and cytokine secretion of CD8+T cell by leaping the CD55/CD59 expression. Interestingly, the therapy integrating anti-CD55/CD59 and anti-PD-1 antibodies evokes a collegial cancer-killing outcome [10]. These findings accentuate the pivotal purpose of ceRNA expression in fine-tuning ICT resistance. Unluckily, the comprehensive analysis exploring the response to immunotherapy especially immune checkpoint blockade (ICB) on ceRNAs in NSCLC is lacking. Further research is warranted to clarify the crosstalk between ceRNA and immune features.

**Figure 1:**
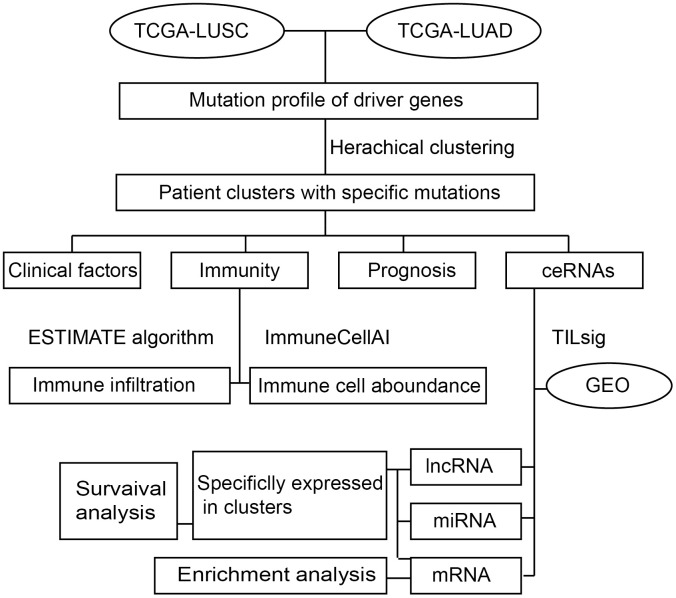
The workflow.

In this study, the transcriptome data and gene mutation profiles of LUAD and LUSC patients were collected from The Cancer Genome Atlas (TCGA) database [11]. With reference to the driver mutations that have been validated in the previous study [12], a hierarchical clustering algorithm was employed to cluster NSCLC patients into subgroups based on driver gene mutation frequencies. Next, survival analysis was applied to investigate the survival risk for NSCLC subgroups. What’s more, we evaluated the immune cell infiltration by using the estimation of stromal and immune cells in malignant tumors using expression data (ESTIMATE) algorithm [13]. The characteristics of the immune microenvironment for each NSCLLC subgroup were estimated by using ImmuneCellAI web tools [14]. Furthermore, we created an immune-related ceRNA signature by using TILsig [15] and validated the efficacy in the GEO dataset. The specific-ceRNAs in NSCLC subgroups were considered as specific markers of the matching NSCLS mutation subgroups. Of course, the survival analysis and function enrichment analysis were also conducted to highlight the role of immune-related ceNRA in NSCLS mutation. The overall design of our study was shown in Figure 1.

## RESULTS

### Somatic mutation profiles of NSCLC

The mutation profile of LUSC contained 158,757 SNP mutations occurring in 18,484 genes in 490 NSCLC samples. Specifically, EGFR mutate in 17 samples, KRAS mutate in 6 samples, 323 samples had TP53 mutations and 54 samples had PIK3CA mutation, and BRAF mutate in 12 samples. The highest mutation frequency in LUSC was TTN, followed by MUC16, CSMD3, TP53, and RYR2. In LUAD, 172,086 mutations occurred in 18,484 genes in 563 samples. According to the mutation frequency, TTN was the most frequently mutated gene and 731 samples had TTN mutation. Next, MUC16 mutated in 470 samples and RYR2 mutate in 396 samples. Mutations in the same gene can occur at different genomic positions, LUSC shared 2418 mutations with LUAD at the same position in the same gene. In addition, the Missense type mutation on LUSC was the most at 59.68%, followed by the Silent type mutation at 21.37%. Missense and Silent mutations in LUAD accounted for 60.79% and 20.42%, respectively. To sum up, the mutation number and mutation type of the two NSCLC subpopulations were similar. It seems that the pathological types had a minor role in determining the driving mutation of NSCLC.

### NSCLC subgroups identified by distinct mutation frequency

**Figure 2:**
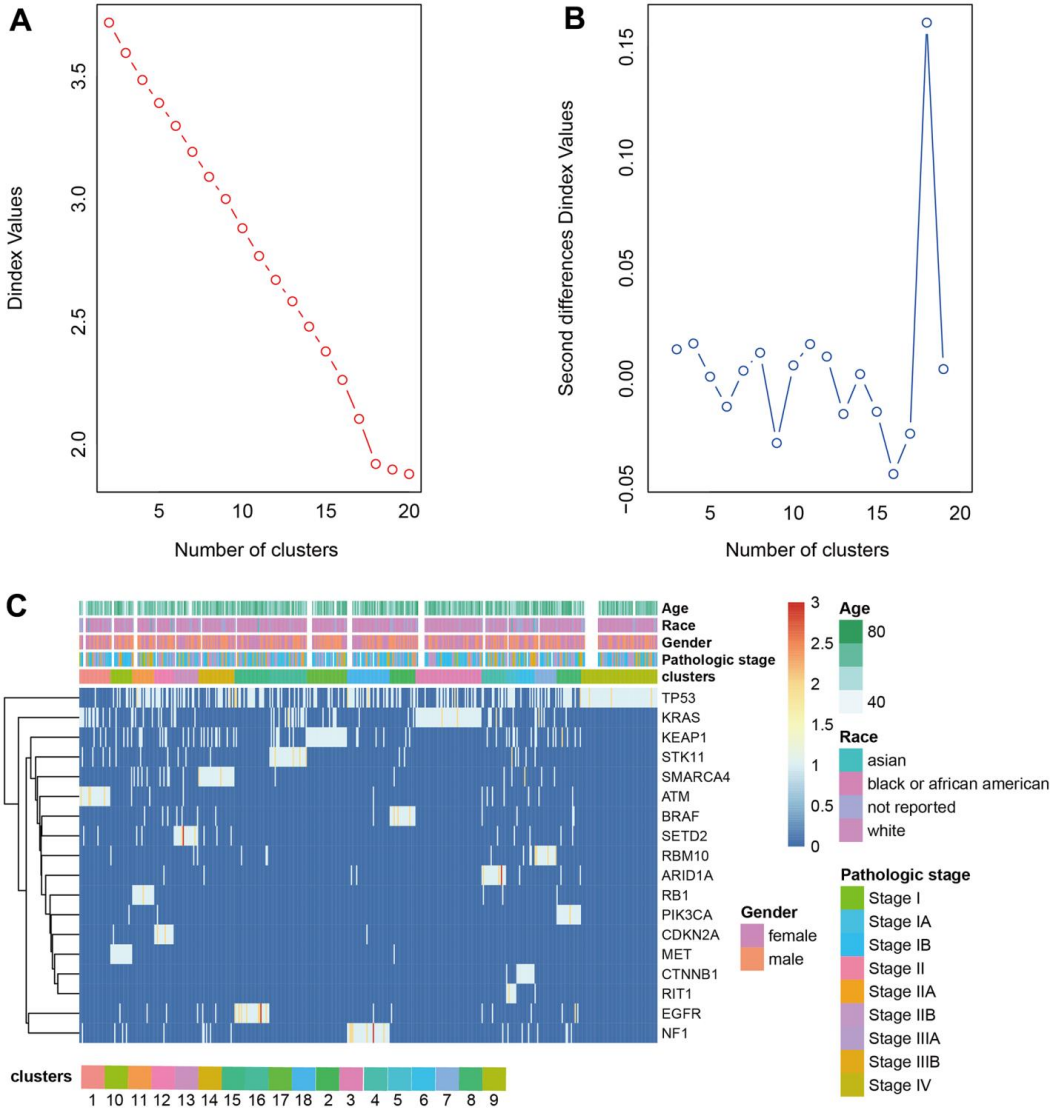
**Clustering analysis LUAD.** (**A**, **B**) The cluster analysis of hierarchical cluster with the optimal parameters. (**C**) The heatmap of 18 clusters containing the clinical features.

**Figure 3:**
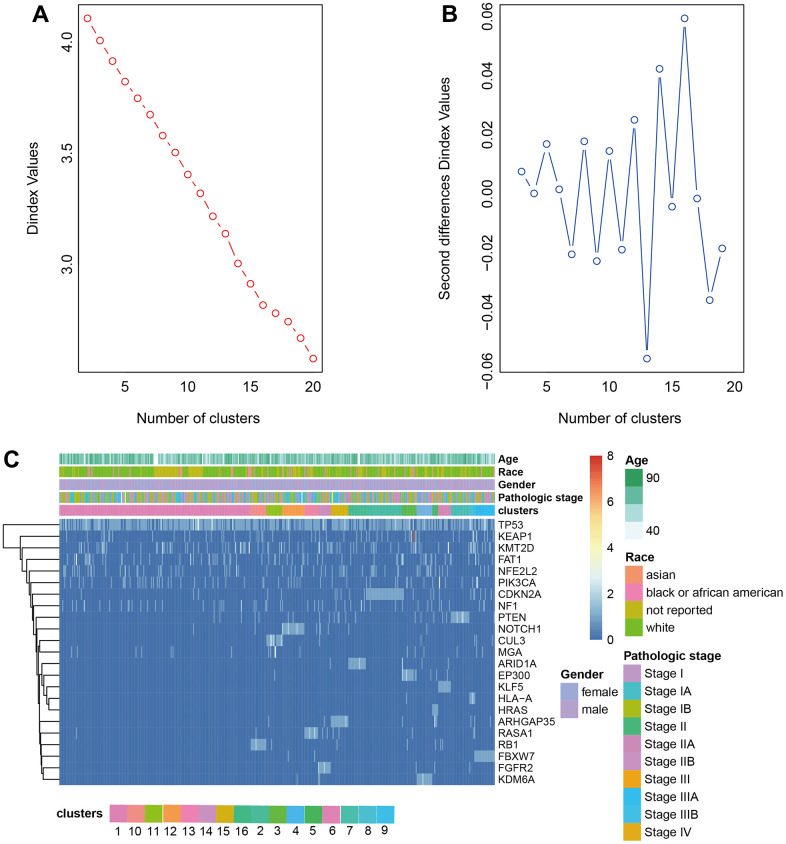
**Clustering analysis LUSC.** (**A**, **B**) The cluster analysis of hierarchical cluster with the optimal parameters. (**C**) The heatmap of 16 clusters containing the clinical features.

The driver genes of NSCLC were obtained from the research of Matthew H. Bailey et al. [12] and the driver gene mutation was extracted from the somatic mutation dataset downloaded from TCGA. The frequency matrix of mutations was scaled to calculate the Euclidean distance between samples to produce the distance matrix. The optimal number of classes was determined by Nbclust. LUAD was divided into 18 clusters and LUSC was divided into 16 clusters by hierarchical clustering (Figures 2A, 2B, 3A, 3B). The heatmap of cluster analysis was displayed in Figures 2C, 3C. We could acquire each cluster-specific gene mutation. For example, among the 16 groups of LUSC, the mutations of cluster 1 were relatively mixed. RB1 mutation was mostly enrichment in cluster 10. MGA gene and CUL3 mutations were Cluster 11-specific. Compared with LUSC, the clustering effect of LUAD was better. Samples containing TP53 mutation and fewer mutations in other genes were clustered as cluster 9, and the other clusters all contained 1 cluster-specific mutation.

### Correlation analysis between clinical characteristics and NSCLC mutation subgroups

The clinical information, including population, gender, aging, and clinical stages, was obtained from TCGA. Correlation analysis uncovered that there is no linear relationship between NSCLC mutation subgroups and clinical features.

### Survival analysis in NSCLC mutation subgroups

**Figure 4:**
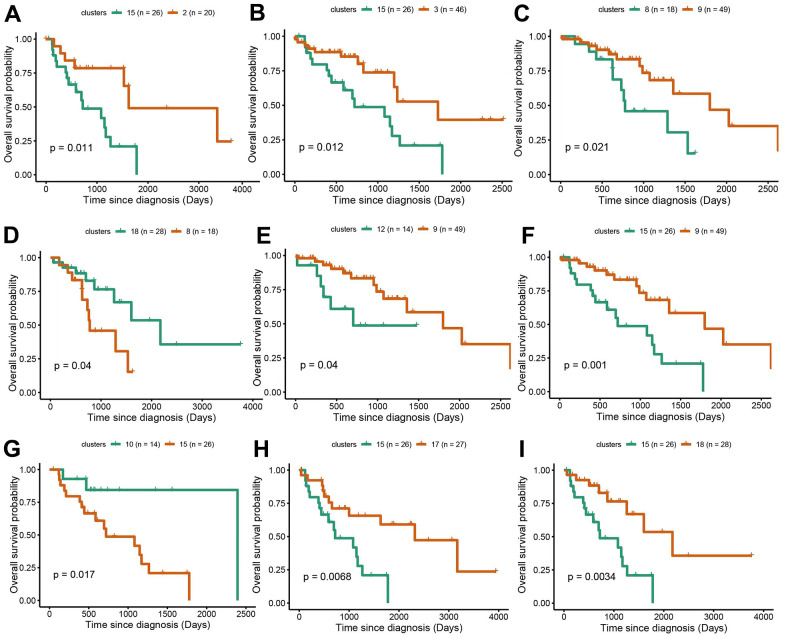
**Survival analysis between subgroups of LUAD.** (**A**–**I**) The survival probabilities between different clusters in LUAD. The statistical analysis was calculated based on log-rank test.

**Figure 5:**
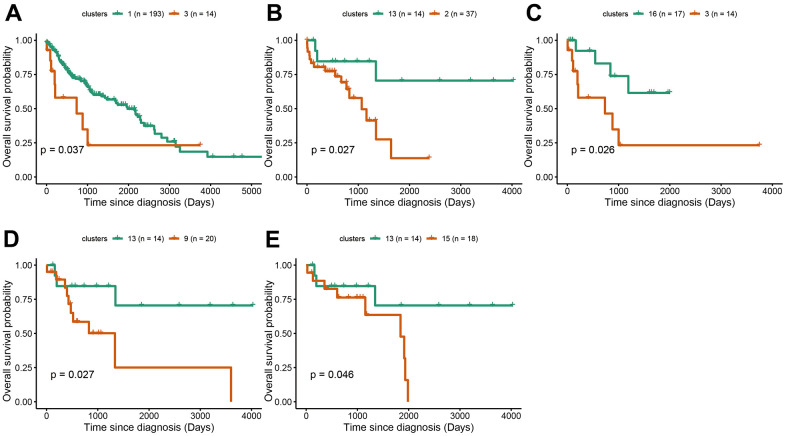
**Survival analysis between subgroups of LUSC.** (**A**–**E**) The survival probabilities between different clusters in LUSC. The statistical analysis was calculated based on log-rank test.

Survival analysis was performed in LUAD and LUSC clusters to examine the influence of cluster-specific gene mutations on NSCLC prognosis. In LUAD, there was a significant survival difference between the following comparisons: cluster1 vs cluster9, cluster5 vs cluster12, cluster6 vs cluster7, cluster7 vs cluster9, cluster8 vs cluster12, cluster9 vs cluster12, cluster14 vs cluster12, cluster17 vs cluster12 (Figure 4A–4H). It was worth noting that cluster9 characterized by TP53 mutation had a better prognosis while cluster12 characterized by CDKN2A mutation, has a poor prognosis. Likewise, in LUSC, we found that there was a significant survival difference between the following comparisons: cluster1 vs cluster3, cluster2 vs cluster13, cluster3 vs cluster16, cluster 9 vs cluster13, and cluster13 vs cluster15 (Figure 5A–5E). We noted that cluster 3 contained EP300 mutation, suggesting that EP300 mutation was positively associated with poor prognosis while cluster 13 characterized by RASA1 mutation showed a good prognosis.

### Analysis of immune microenvironment features in NSCLC mutation subgroups

**Figure 6:**
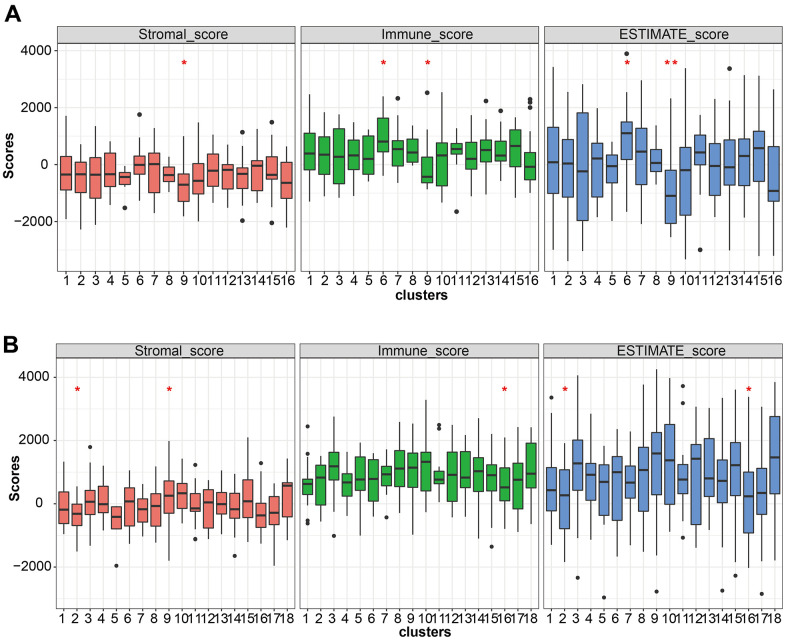
**Immune cell infiltration analysis.** (**A**, **B**) The stromal score, immune score, and estimate score pattern estimated by the ESTIMATE algorithm in LUSC and LUAD.

**Figure 7:**
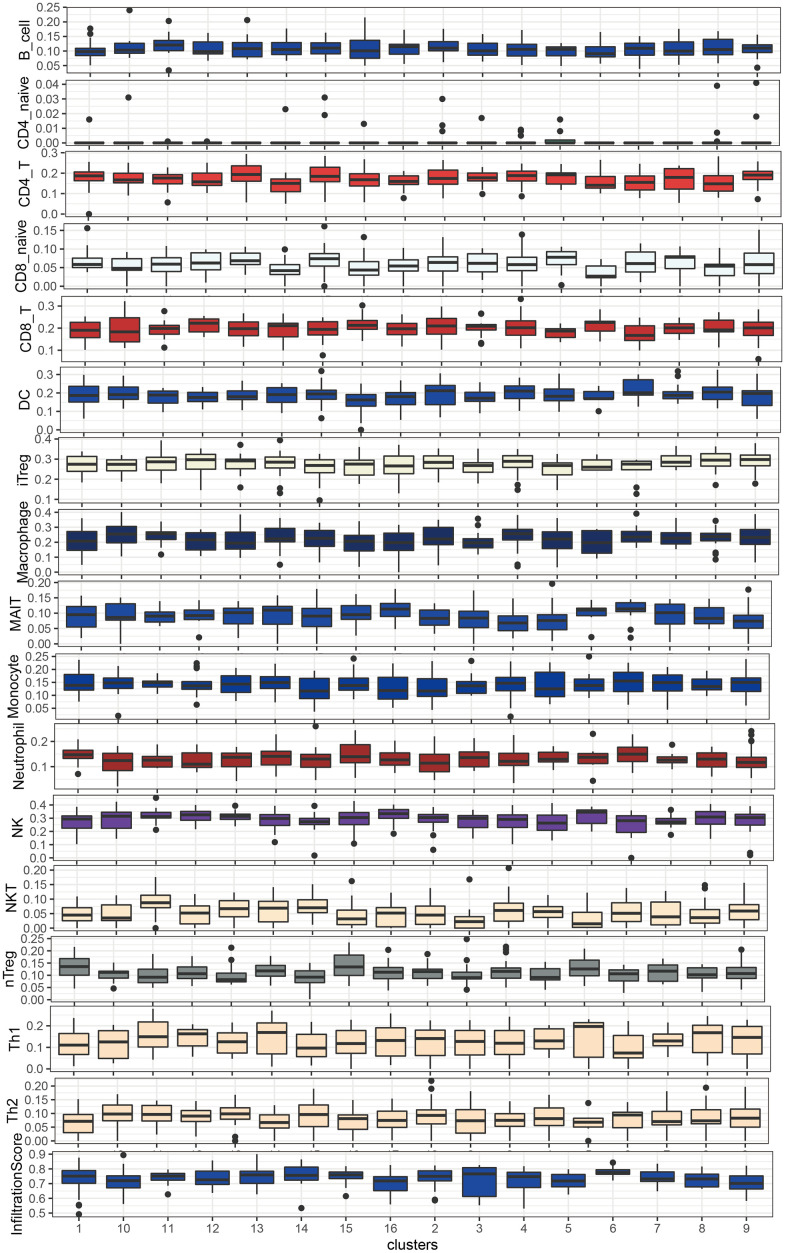
Immune cell abundance in LUSC subgroups by ImmuneCellAI.

The ESTIMATE algorithm was used to calculate the immune score (immune cell), stromal score (stromal cell), and estimate score (tumor purification) to evaluate the degree of immune cell infiltration between LUAD and LUSC mutation clusters (Figure 6A, 6B). The proportion of T cells with distinct functional phenotypes was predicted with ImmuneCellAI and we calculated the infiltration score, an index of the immune cell infiltration level in LUSC (Figure 7). Cluster9 in LUAD carried abundant immune cell infiltration, suggesting that cluster 9 had better immunity and patients in cluster 9 were suitable for immune therapy.

### Identification of immunity-related ceRNA

From the GSE28490 dataset, the immune-related mRNA and miRNA signatures were obtained according to the preliminary criteria of TILs. According to the previous study, we collected the immune-related lncRNA lists [15]. A total of 146 mRNAs, 20 miRNAs, and 92 lncRNAs were enrolled in our subsequent analysis.

**Figure 8:**
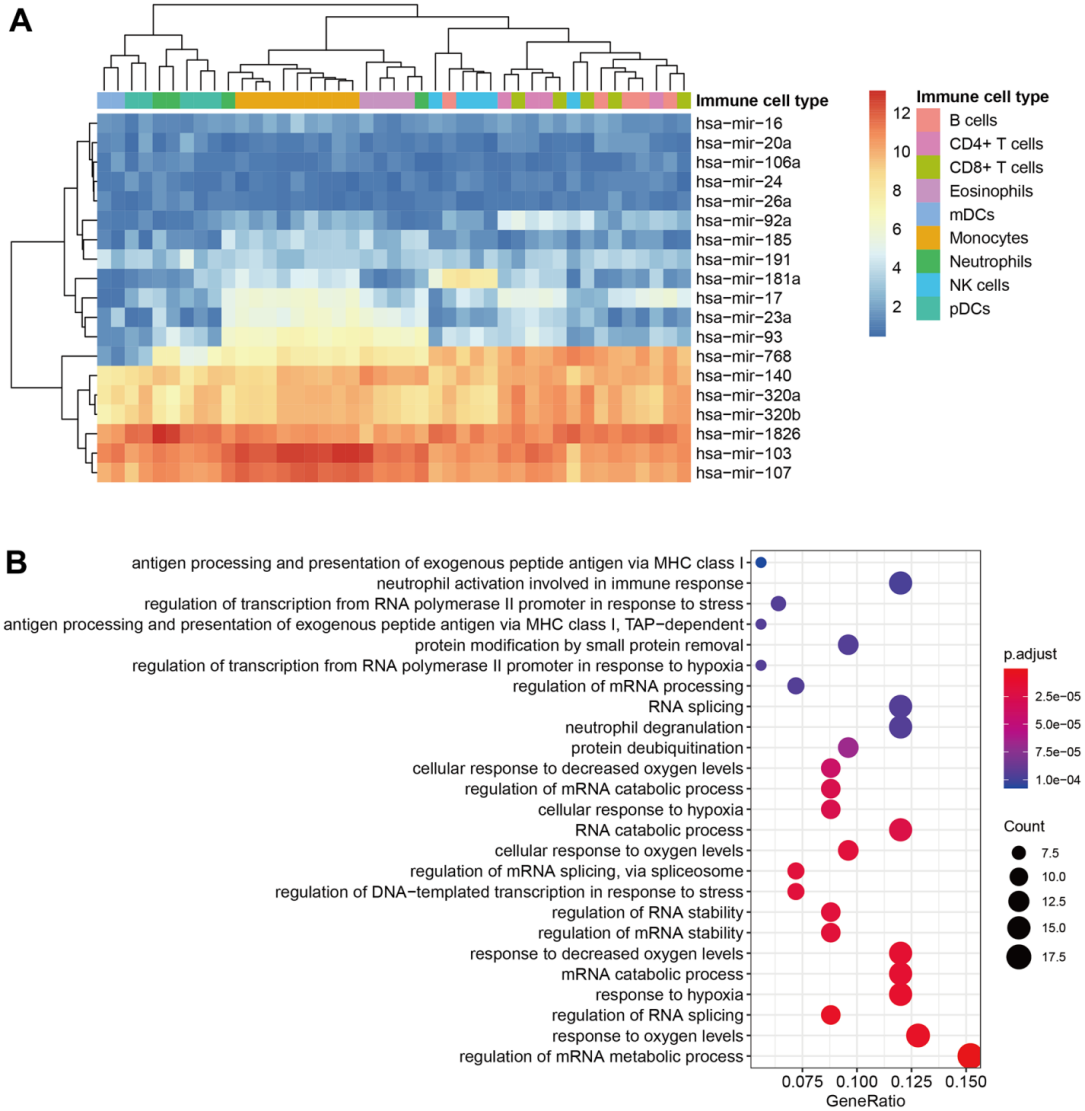
**Identification of immune-related ceRNAs.** (**A**) The heatmap of selected miRNA in immune cells. (**B**) The KEGG analysis of selected mRNA.

The cluster-specific ceRNAs were screened out according to the differential expression of selected ceRNA signatures in the immune cell of each cluster and they were functionally related to the cluster-specific mutation. We found that hsa-miR-768, hsa-miR-140, hsa-miR-320, hsa-miR-1826, hsa-miR-103, and hsa-miR-107 were enhanced in immune cell (Figure 8A). KEGG analysis indicated that the cluster-specific mRNAs were mainly engaged in RNA metabolism-associated pathways (Figure 8B).

### Survival analysis of immune-related ceRNAs in NSCLC mutation subgroups

To verify the cluster-specificality of the identified ceRNAs, survival analysis was performed in NSCLC mutation subgroups. Survival analysis based on the unclustered NSCLC samples indicated that there was not a significant association at the overall level while survival analysis based on the NSCLC mutation subgroups showed that ceRNA was significantly associated with survival. In LUAD, has-miR-191 was highly expressed in cluster12 while has-mir-185 was highly expressed in cluster9 and associated with a better prognosis. Furthermore, ACTB, ACTR2, ADAR, ADD3, ARHGAP, ARPC2, ATP6V0E1, BCLAF1, BTG1, CAPZB, CCNI, CCNL1, CD53, CDC42SE2, CELF2, CNBP, CORO1A, CXCR4, EIF1, EIF4G2, ELOVL5, FTL, GABARAP, GDI2, GHITM, GNB1, H2AFY, HCLS1, HIF1A were significantly differentially expressed between interested subgroups.

**Figure 9:**
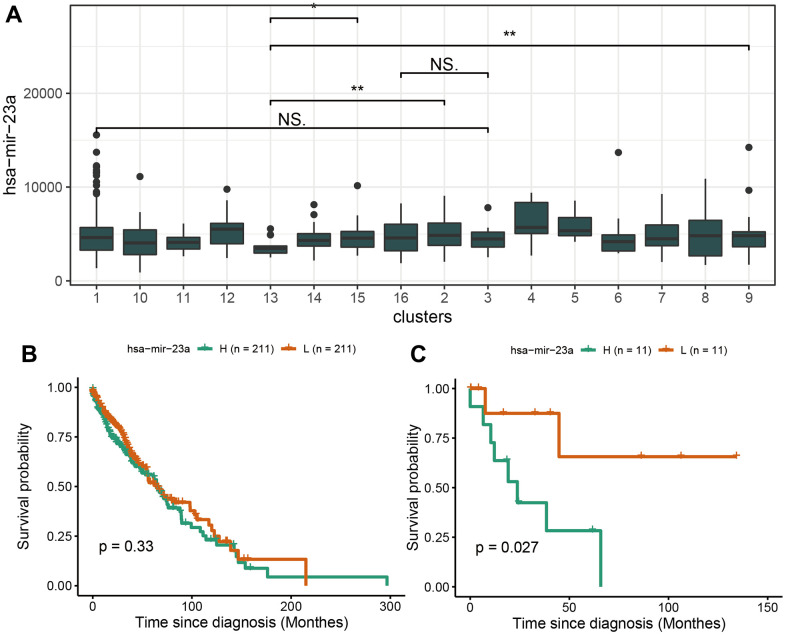
**Survival analysis of hsa-miR-23a.** (**A**) The expression profile of hsa-miR-23a in 16 clusters of LUSC. (**B**) The survival analysis of hsa-miR-23a in overall LUSC samples. (**C**) The survival analysis of hsa-miR-23a in RASA1-mutation cluster of LUSC.

In LUSC, has-miR-17 was highly expressed in cluster3 and associated with a poor prognosis. In LUSC, hsa-miR-23a was low-expressed in cluster13 characterized by the RASA1 mutation (Figure 9A). The analysis found that the expression of hsa-miR-23a was not highly correlated with survival in the overall LUSC sample while in cluster 13 with RASA1 mutation, has-miR-23a was reduced in cluster13 and associated with a good prognosis (Figure 9B, 9C).

## DISCUSSION

In the current study, we divided LUSC samples into 16 clusters and LUAD samples into 18 clusters, which were characterized by unique mutation patterns according to the frequency of tumor driver mutations profiles in LUSC and LUAD, respectively. Each subgroup has its signature mutation. The distinct NSCLS mutation subgroups indicated the complexity and diversity of NSCLC gene variations. Survival analysis of pairwise comparison showed that some subtypes had significantly higher survival rates, and subgroup-specific gene mutation may be a potential prognosis biomarker in matching NSCLC subgroups. For example, Ras p21 protein activator 1 (RASA1), specifically mutated in cluster13 in LUSC, is a regulator of Ras GDP and GTP dynamic conversion. Increasing evidence has pinpointed that RASA1 is involved in numerous cancer-related physiological processes such as angiogenesis, cell proliferation, and apoptosis [16]. Previous research has reported that hsa-miR-182 suppresses cell proliferation and viability by recognizing the 3’UTR region of RASA1 in LUSC [17]. Here, our data highlighted the functional govern of RASA1 in LC survival outcomes. Taken together, RASA1 mutation was a favorable factor in LUSC prognosis.

Similarly, CDKN2A, an important tumor-driving gene, is specifically mutated in cluster 12 in LUAD. Stanley I. Gutiontov et al. revealed that CDKN2A negatively impacts clinical outcomes in advanced NSCLC treated with immune checkpoint blockade therapy and the survival analysis also suggested NSCLC patients carrying CDKN2A-mutation have a poor prognosis [18]. Our research indicated that, compared with cluster 8, cluster 9, cluster 14, and cluster 17, the survival time of cluster 12 with CDKN2A mutation was shorter, implying that CDKN2A mutation was an unfavorable risk factor in LUAD survival. Correcting the CDKN2A mutation can be an alternative approach to improving LUAD prognosis.

Further, the ESTIMATE method and the infiltration algorithm from ImmuneCellAI were used to estimate the immune infiltration of each cluster. The immune cell infiltration was reduced in cluster 9. Cluster 9 in LUSC mainly contained the FBXW7 mutation. Mounting data have suggested that FBW7 decreases the stability of PD-1 by inhibiting the K48-linked de-polyubiquitination of PD-1, which confers the tumor microenvironment too sensitive to response to PD-1 blockade therapy by recruiting the tumor-infiltrating cytotoxic T cell in NSCLC [19]. Taken together, targeting FBXW7 shows great promise in improving the ICT effect.

The scores of neutrophil and regulatory T cells of cluster 12 characterized by CDK2NA mutation were significantly lower than in other clusters. This may partly explain the cause of the worse prognosis. In fact, CDKN2A (encoding p16INK4A) exerts a far-reaching tumor-inhibition purpose by enhancing the KRAS signaling pathways activity in accelerating the malignant progression of cancer. The small molecule inhibitors targeting CDK4 and CDK6 recuperate the impaired tumor suppression function induced by p16INK4A mutation, which revives the tumor growth in pancreatic ductal adenocarcinoma (PDAC) [20]. Besides, Kaplan Meier curves of immune-related ceRNAs in LUSC showed that has-miR-23a was not significantly associated with patient survival in the overall samples, but was significantly associated with survival in RASA1 mutation samples, indicating that there may be specific mutations in clusters containing different marker mutations and ceRNA marker molecules.

## MATERIALS AND METHODS

### Data source

The LUSC and LUAD mutation data and mRNA, miRNA, lncRNA expression profile data were downloaded from TCGA using TCGAbiolinks (2.26.0) [21] R package, including 504 patients in LUSC and 594 patients in LUAD. We downloaded the mRNA expression data of 9 immune cell lines of GSE28490 [22] directly from the website (48 samples * 20368 probes), the miRNA expression data of 9 immune cell lines from GSE28487 [22] (48 samples * 7816 miRNA probes). The immune-related mRNAs and miRNAs were extracted from the previous study [23]. The immune-lncRNA signature was acquired according to a previous study [15]. The immune-related core molecules of mRNA and miRNA are calculated from Gene Expression Omnibus (GEO) database [24].

### Clustering analysis based on mutation profiles

The driver genes of LUSC and LUAD were obtained from the research of Matthew H. Bailey et al. [12] and the driver gene mutation was extracted from the somatic mutation dataset of TCGA. Each gene was counted according to the distribution of mutations in the sample. The frequency matrix of mutations was scaled to calculate the Euclidean distance between samples to produce the distance matrix. Then hierarchical clustering was used to cluster the samples into different groups. The optimal number of clusters was determined with R package Nbclust (3.0.1) [25]. LUSC was clustered into 16 clusters, and LUAD was clustered into 18 clusters.

### Immune infiltration analysis

ESTIMATE algorithm, a method that uses gene expression signatures to infer the fraction of stromal and immune cells, was used to detect the inflating scores of tumor and immune cells scores in the tumor environment (https://bioinformatics.mdanderson.org/public-software/estimate/). Stromal cell score, immune cell score, and overall estimate score were calculated by input expression matrix of LUAD and LUSC to the web tool. ImmuCellAI (Immune Cell Abundance Identifier) was used to estimate the abundance of 24 immune cells based on the gene expression dataset including RNA-Seq and microarray data (http://bioinfo.life.hust.edu.cn/ImmuCellAI#!/). The expression profiles of LUSC and LUAD were obtained from TCGA.

### Survival analysis

The expression profiles of ceRNAs and related clinical information of LUAD and LUSC samples were acquired from the TCGA database. To identify the prognosis-related ceRNAs, survival analysis based on the Cox model was used to estimate the survival risk of patients in different groups. For a specific ceRNA, patients were divided into two groups according to the median expression of the ceRNA. Then Kaplan-Meier curve analysis was performed with the “Survival” package in R and the p-value between the two groups was also calculated based on log-tank test. The p.value < 0.05 were considered significant.

### Function enrichment analysis

To explore the potential biological functions and pathways of immune-related signature mRNAs, we performed Gene Ontology (GO) [26] and Kyoto Encyclopedia of Genes and Genomes (KEGG) [27] pathway analysis on 146 immune-related genes (Supplementary Table 2) using the “ClusterProfiler” package in R [28]. GO terms and KEGG pathways with p < 0.05 were considered significantly enriched.

### Availability of data and materials

Five processed data have been supplied as the Supplementary Tables 1–5. The code script was supplied (Supplementary File 1). The other datasets used and/or analyzed in the present study are available from the corresponding author on reasonable request.

## Supplementary Material

Supplementary Table 1

Supplementary Table 2

Supplementary Table 3

Supplementary Table 4

Supplementary Table 5

Supplementary File 1
