# Supplementary material for: Identification of prognosis value and immune microenvironment features of ceRNAs in NSCLC with distinct gene mutation
Source: Aging (Albany NY). 2023 Jun 26;15(12):5873–86. doi: 10.18632/aging.204846 (PMC10333077; doi:10.18632/aging.204846)
Supplement: Supplementary File 1 [file aging-15-204846-s006.docx]

Supplementary File 1. The code script of the bioinformatical analysis.

#01-download data from TCGA

library(TCGAbiolinks)

library(tidyverse)

library(SummarizedExperiment)

setwd("01.data")

#mRNA profile

count_lusc <- GDCquery(project = "TCGA-LUSC",

data.category = "Transcriptome Profiling",

data.type = "Gene Expression Quantification",

workflow.type = "HTSeq - Counts")

)

GDCdownload(count_lusc)

exp_count_lusc <- GDCprepare(query = count_lusc,save = TRUE, save.filename = "count_lusc.rda")

fpkm_lusc <- GDCquery(project = "TCGA-LUSC",

data.category = "Transcriptome Profiling",

data.type = "Gene Expression Quantification",

workflow.type = "HTSeq - FPKM-UQ")

GDCdownload(fpkm_lusc)

exp_fpkm_lusc <- GDCprepare(query = fpkm_lusc,save = TRUE, save.filename = "fpkm_lusc.rda")

count_luad <- GDCquery(project = "TCGA-LUAD",

data.category = "Transcriptome Profiling",

data.type = "Gene Expression Quantification",

workflow.type = "HTSeq - Counts")

)

GDCdownload(count_luad)

exp_count_luad <- GDCprepare(query = count_luad,save = TRUE, save.filename = "count_luad.rda")

fpkm_luad <- GDCquery(project = "TCGA-LUAD",

data.category = "Transcriptome Profiling",

data.type = "Gene Expression Quantification",

workflow.type = "HTSeq - FPKM-UQ")

GDCdownload(fpkm_luad)

exp_fpkm_luad <- GDCprepare(query = fpkm_luad,save = TRUE, save.filename = "fpkm_luad.rda")

#miRNA

mirna_lusc <- GDCquery(project = "TCGA-LUSC",

experimental.strategy = "miRNA-Seq",

data.category = "Transcriptome Profiling",

# barcode = c("TARGET-20-PATDNN","TARGET-20-PAPUNR"),

data.type = "miRNA Expression Quantification")

GDCdownload(mirna_lusc)

mirna_lusc <- GDCprepare(query = mirna_lusc,save = TRUE, save.filename = "mirna_lusc.rda")

mirna_luad <- GDCquery(project = "TCGA-LUAD",

experimental.strategy = "miRNA-Seq",

data.category = "Transcriptome Profiling",

# barcode = c("TARGET-20-PATDNN","TARGET-20-PAPUNR"),

data.type = "miRNA Expression Quantification")

GDCdownload(mirna_luad)

mirna_luad <- GDCprepare(query = mirna_luad,save = TRUE, save.filename = "mirna_luad.rda")

#mutation profile

maf_lusc <- GDCquery_Maf("LUSC", pipelines = "muse")

saveRDS(maf_lusc,file="mal_lusc.rds")

maf_lusc <-readRDS("mal_lusc.rds")

maf_luad <- GDCquery_Maf("LUAD", pipelines = "muse")

saveRDS(maf_luad,file="mal_luad.rds")

maf_luad <-readRDS("mal_luad.rds")

#clinic information

clin_lusc <- GDCquery_clinic("TCGA-LUSC", "clinical")

write.table(clin_lusc,"clin_lusc.tsv",sep = '\t',row.names = F)

clin_lusc <- read.table("clin_lusc.tsv",sep = '\t',header = T)

clin_luad <- GDCquery_clinic("TCGA-LUAD", "clinical")

write.table(clin_luad,"clin_luad.tsv",sep = '\t',row.names = F)

clin_luad <- read.table("clin_luad.tsv",sep = '\t',header = T)

#get sample type

samplesDown <- getResults(count_lusc,cols=c("cases"))

saveRDS(samplesDown,file = "lusc_sampledown.rds")

dataSmTP <- TCGAquery_SampleTypes(barcode = samplesDown,

typesample = "TP")

dataSmNT <- TCGAquery_SampleTypes(barcode = samplesDown,

typesample = "NT")

#02-hc-cluster

library(tidyverse)

library(reshape2)

#mutation file: maf_lusc, maf_luad

#clinical file: clin_lusc, clin_luad

#public markers

markers_pub<- c("EGFR","ALK","KARS","TP53","PIK3CA","BRAF","MET","NF1")

markers_pub <- c("EGFR","BRAF","ROS1","FGFR1","KRAS","MET","HER2","NRTK","RET","ALK",

"NRG1","TP53","PTEN","PIK3CA")

library(openxlsx)

setwd("analysis/01.data")

markers3 <- read.xlsx("NIHMS948705-supplement-8.xlsx",sheet = 2,colNames = T)

markers3%>%

filter(X2=="LUSC")->marker.lusc

marker.lusc$`Table.S1:.Final.gene.consensus.list..Related.to.Figure.2.`->marker.lusc.1

markers3%>%

filter(X2=="LUAD")->marker.luad

marker.luad$`Table.S1:.Final.gene.consensus.list..Related.to.Figure.2.`->marker.luad.1

marker.lung <- unique(c(marker.lusc.1,marker.luad.1))

#path

setwd("analysis/02.cluster_by_mutation")

unique(maf_lusc$Tumor_Sample_Barcode)[!(grepl("-01A-",unique(maf_lusc$Tumor_Sample_Barcode)))]

length(unique(maf_lusc$Hugo_Symbol))

#1

quantile(table(maf_lusc$Hugo_Symbol))

#2

maf_lusc%>%

dplyr::select(Hugo_Symbol,Chromosome,Start_Position,End_Position,Strand,

Variant_Classification,

Tumor_Sample_Barcode)%>%

filter(Hugo_Symbol %in% marker.lusc.1)%>%

mutate(var_id = paste0(Hugo_Symbol,"_",

Chromosome,"_",

Start_Position,"_",

End_Position,"_",

Strand))->maf_lusc_brief

dim(maf_lusc_brief)

maf_luad%>%

dplyr::select(Hugo_Symbol,Chromosome,Start_Position,End_Position,Strand,

Variant_Classification,

Tumor_Sample_Barcode)%>%

filter(Hugo_Symbol %in% marker.luad.1)%>%

mutate(var_id = paste0(Hugo_Symbol,"_",

Chromosome,"_",

Start_Position,"_",

End_Position,"_",

Strand))->maf_luad_brief

dim(maf_luad_brief)

maf_lusc%>%

rbind(maf_luad)%>%

select(Hugo_Symbol,Chromosome,Start_Position,End_Position,Strand,

Variant_Classification,

Tumor_Sample_Barcode)%>%

filter(Hugo_Symbol %in% marker.lung)%>%

mutate(var_id = paste0(Hugo_Symbol,"_",

Chromosome,"_",

Start_Position,"_",

End_Position,"_",

Strand))->maf_lung_brief

dim(maf_lung_brief)

#table(maf_lusc_brief$Hugo_Symbol)[order(table(maf_lusc_brief$Hugo_Symbol),decreasing = T)][1:50]->features

#write.table(as.data.frame(features),file = "selected_mutation_genes.csv",sep = ',')

#features <- read.table("selected_mutation_genes.csv",sep = ',')

#table(maf_lusc_brief$var_id)

maf_lusc_brief%>%

dplyr::select(Tumor_Sample_Barcode,Hugo_Symbol)%>%

mutate(count=1)->test

dcast(test,Hugo_Symbol~Tumor_Sample_Barcode)->maf_lusc_df

rownames(maf_lusc_df) <-maf_lusc_df$Tumor_Sample_Barcode

maf_luad_brief%>%

dplyr::select(Tumor_Sample_Barcode,Hugo_Symbol)%>%

mutate(count=1)->test

dcast(test,Hugo_Symbol~Tumor_Sample_Barcode)->maf_luad_df

rownames(maf_luad_df) <-maf_luad_df$Tumor_Sample_Barcode

maf_lung_brief%>%

select(Tumor_Sample_Barcode,Hugo_Symbol)%>%

mutate(count=1)->test

dcast(test,Hugo_Symbol~Tumor_Sample_Barcode)->maf_lung_df

rownames(maf_lung_df) <-maf_lung_df$Tumor_Sample_Barcode

maf_lusc_df[is.na(maf_lusc_df)] <-0

maf_lusc_df_tmp <- apply(maf_lusc_df[,-1], 2,as.numeric)

rownames(maf_lusc_df_tmp) <-maf_lusc_df$Hugo_Symbol

maf_luad_df_tmp <- apply(maf_luad_df[,-1], 2,as.numeric)

rownames(maf_luad_df_tmp) <-maf_luad_df$Hugo_Symbol

maf_lung_df_tmp <- apply(maf_lung_df[,-1], 2,as.numeric)

rownames(maf_lung_df_tmp) <-maf_lung_df$Hugo_Symbol

saveRDS(maf_lusc_df_tmp,"maf_lusc.df.rds")

maf_lusc_df_tmp <- readRDS("maf_lusc.df.rds")

dim(maf_lusc_df_tmp)

saveRDS(maf_luad_df_tmp,"maf_luad.df.rds")

maf_lusc_df_tmp <- readRDS("maf_luad.df.rds")

head(maf_luad_df_tmp)[,1:6]

#

library(pheatmap)

maf_lusc_df_tmp->hp_df

pdf(file = "lusc/hp.drvergene.mutfreq.pdf",width = 8,height = 6)

pheatmap(hp_df)

dev.off()

maf_luad_df_tmp->hp_df

pdf(file = "luad/hp.drvergene.mutfreq.pdf",width = 8,height = 6)

pheatmap(hp_df)

dev.off()

#

#maf <- scale(t(maf_lusc_df_tmp))

#

maf <- scale(t(maf_luad_df_tmp))

hc2<-hclust(dist(maf),method = "single")

pdf("hc.single.pdf",width = 8,height = 12)

plot(hc2,hang = -0.01,cex=0.7)

dev.off()

library(NbClust)

nc<-NbClust(maf,distance = "euclidean",

min.nc = 2,max.nc = 20,

method = "ward.D2")

table(nc$Best.nc[1,])

barplot(table(nc$Best.nc[1,]))

clusters<-cutree(hc,k=18)

table(clusters)

write.table(as.data.frame(clusters),file = "luad/m3.clusters.csv",sep = ',')

pdf("hc.m3.cluster.pdf",width = 8,height = 4)

plot(hc,cex=0.7,hang=-0.01)

rect.hclust(hc,k=5,border="red")

dev.off()

clusters <- read.table("luad/m3.clusters.csv",sep = ',')

clusters$barcode <- rownames(clusters)

table(clusters$clusters)

#for combine

clusters$clusters <- as.character(clusters$clusters)

clusters%>%

mutate(cancer_type = ifelse(barcode %in% maf_lusc$Tumor_Sample_Barcode,"LUSC","LUAD"))%>%

select(-barcode)->clusters

clusters[order(clusters$clusters),]->clusters

clusters%>%

dplyr::select(-barcode)->clusters

pdf("luad/hp.gcluster.pdf",width = 8,height = 6)

pheatmap(maf_luad_df_tmp[,rownames(clusters)],annotation_col = clusters,cluster_cols = F)

dev.off()

pdf("luad/hp.acluster.pdf",width = 8,height = 7)

pheatmap(maf_luad_df_tmp[,rownames(clusters)],annotation_col = clusters,cluster_cols = T)

dev.off()

#03-cluster-survival

setwd("analysis/02.cluster_by_mutation")

clusters <- read.table(file = "lusc/m3.clusters.csv",sep = ',')

clusters$clusters<- as.character(clusters$clusters)

clusters$sample_id <- rownames(clusters)

clusters[order(clusters$clusters),]->clusters

clusters%>%

select(-sample_id)->clusters

clusters["submitter_id"] <- str_sub(rownames(clusters),1,12)

setwd("/analysis/03.cluster_clincal")

maf_lusc%>%

select(Hugo_Symbol,Tumor_Sample_Barcode)%>%

mutate(count=1)->maf_lusc_brief

maf_lusc_brief%>%

mutate(clusters=clusters[maf_lusc_brief$Tumor_Sample_Barcode,])->maf_cluster

table(maf_cluster$clusters)

#mutation

#as.data.frame(table(maf_cluster$Hugo_Symbol,maf_cluster$clusters))-> mut_clu

#dcast(mut_clu,Var1~Var2,value.var = "Freq")->mut_clu

#rownames(mut_clu)<-mut_clu$Var1

view(head(maf_lusc))

dcast(maf_lusc_brief,Hugo_Symbol~Tumor_Sample_Barcode) -> mut_df

rownames(mut_df) <- mut_df$Hugo_Symbol

head(mut_df)[,1:6]

dim(mut_df)

library(pheatmap)

mut_df[rowSums(mut_df[,-1])>20,]->mut_df_filt

dim(mut_df_filt)

mut_df -> mut_df_filt

pdf("hp.m3.clu.pdf",width = 6,height = 6)

pheatmap(mut_df_filt[marker.lusc.1,rownames(clusters)],

annotation_col = clusters,

cluster_cols=F)

dev.off()

pheatmap()

#clinical

clin_lusc

colnames(clin_lusc)

clusters$submitter_id <- substr(rownames(clusters),1,12)

clin_lusc%>%

select(submitter_id,ajcc_pathologic_stage)%>%

right_join(clusters)->clin_stage

dim(clin_stage)

head(clin_stage)

clin_luad%>%

select(submitter_id,ajcc_pathologic_stage)%>%

right_join(clusters)->clin_stage

ggplot(clin_stage,aes(clusters,fill=ajcc_pathologic_stage))+

geom_bar(stat="count")+

theme_bw()->p

ggsave(filename = "m3.stage.cluster.pdf",plot = p, width = 6,height = 6)

#survival

data.frame(submitter_id = substr(colnames(maf_lusc_df_tmp),1,12),

clusters = ifelse(maf_lusc_df_tmp[i,]>0,1,0))->clusters

setwd("analysis/01.data")

clin_lusc <- read.table("clin_lusc.tsv",sep = '\t',header = T)

clin_lusc%>%

select(submitter_id,days_to_last_follow_up,days_to_death,vital_status)%>%

left_join(clusters)->sur_df

clin_luad%>%

select(submitter_id,days_to_last_follow_up,days_to_death,vital_status)%>%

right_join(clusters)->sur_df

sur_df$days_to_death[is.na(sur_df$days_to_death)] <- 0

sur_df$days_to_last_follow_up[is.na(sur_df$days_to_last_follow_up)] <- 0

sur_df$days=as.numeric(sur_df$days_to_death)+as.numeric(sur_df$days_to_last_follow_up)

#sur_df$days=round(sur_df$days/30,2)

1library(survminer)

library(survival)

plot_2g_sur <- function(g1,g2,sur_df){

sur_df%>%

filter(clusters %in% c(g1,g2))->sur_df_clu

TCGAanalyze_survival(sur_df_clu,

clusterCol="clusters",

legend = "clusters",

risk.table = FALSE,

conf.int = FALSE,

color = c("Dark2"),

#main = gene_symbol,

xlab = "Time since diagnosis (Days)",

dpi=1200,

height = 4, width = 5,

#dpi = 300,

filename = paste0("luad/m3.clu.",g1,".",g2,".survival.pdf"))

}

plot_2g_sur("1","2",sur_df)

...

plot_2g_sur("17","18",sur_df)

#04-TILsig-ceRNA

#TILSig

#immune-related mRNA

setwd("analysis/01.data/GEO-immune-subset")

exp<- read.table("GSE28490_series_matrix.d.txt",header = T)

soft <- readr::read_tsv("GSE28490_family.soft.d1.txt",col_names = T)

soft%>%

select(ID,`Gene Symbol`)->soft_b

cell_type <- read.table("GSE28490.cell_type")

colnames(cell_type) <- colnames(exp)

data.frame(t(cell_type)[-1,])->celltype

for(ct in unique(celltype$t.cell_type...1...)){

ct = unique(celltype$t.cell_type...1...)[9]

sample_list <- rownames(celltype)[celltype$t.cell_type...1...==ct]

exp[,c("ID_REF",sample_list)]%>%

left_join(soft_b,by = c("ID_REF" = "ID"))%>%

filter(!is.na(`Gene Symbol`))%>%

select(-ID_REF)->exp_tmp

exp_tmp$exp_mean <- apply(exp_tmp[,1:length(exp_tmp)-1],1,mean)

exp_tmp[order(exp_tmp$exp_mean,decreasing = T),]->sig_mRNA

sig_mRNA[1:floor(nrow(exp_tmp)*0.05),]->df_ct9

}

intersect(df_ct1$`Gene Symbol`,c("PTPRC"))

Reduce(intersect,list(v1 = df_ct1$`Gene Symbol`,

v2 = df_ct2$`Gene Symbol`,

v3 = df_ct3$`Gene Symbol`,

v4 = df_ct4$`Gene Symbol`,

v5 = df_ct5$`Gene Symbol`,

v6 = df_ct6$`Gene Symbol`,

v7 = df_ct7$`Gene Symbol`,

v8 = df_ct8$`Gene Symbol`,

v9 = df_ct9$`Gene Symbol`))->immun.mRNA

setwd("analysis/04.TILsig-ceRNA")

write.table(as.data.frame(immun.mRNA),file = "mRNA.signature",sep=',')

#immune-related miRNA

exp_mi<- read.table("GSE28487_series_matrix.d.txt",header = T)

soft_mi <- readr::read_tsv("GSE28487_family.soft.txt",col_names = F)

soft_mi%>%

select(X1,X8)->soft_mi_b

cell_type_mi <- read.table("GSE28487.cell_type")

colnames(cell_type_mi) <- colnames(exp_mi)

data.frame(t(cell_type_mi)[-1,])->celltype_mi

for(ct in unique(celltype_mi$t.cell_type_mi...1...)){

ct = unique(celltype_mi$t.cell_type_mi...1...)[1]

sample_list <- rownames(celltype_mi)[celltype_mi$t.cell_type_mi...1...==ct]

exp_mi[,c("ID_REF",sample_list)]%>%

left_join(soft_mi_b,by = c("ID_REF" = "X1"))%>%

filter(!is.na(X8))%>%

select(-ID_REF)->exp_mi_tmp

exp_mi_tmp$exp_mean <- apply(exp_mi_tmp[,1:length(exp_mi_tmp)-1],1,mean)

exp_mi_tmp[order(exp_mi_tmp$exp_mean,decreasing = T),]->sig_miRNA

sig_miRNA[1:floor(nrow(exp_mi_tmp)*0.05),]->dfmi_ct1

}

Reduce(intersect,list(v1 = dfmi_ct1$X8,

v2 = dfmi_ct2$X8,

v3 = dfmi_ct3$X8,

v4 = dfmi_ct4$X8,

v5 = dfmi_ct5$X8,

v6 = dfmi_ct6$X8,

v7 = dfmi_ct7$X8,

v8 = dfmi_ct8$X8,

v9 = dfmi_ct9$X8))->immun.miRNA

setwd("C:/Users/FuXin/OneDrive/FU/LiKe/LungCancer-202112-202206/analysis/04.TILsig-ceRNA")

write.table(as.data.frame(immun.miRNA),file = "miRNA.signature",sep=',')

#lncRNA

library(openxlsx)

immun.linc <- read.xlsx("jitc-2019-000110supp001-lnc-pubmed.xlsx",sheet = 1,startRow = 1,colNames = T)

write.table(as.data.frame(immun.linc),file = "lncRNA.signature",sep=',')

library(pheatmap)

head(exp)[,1:6]

head(soft_b)

soft_b%>%

filter(`Gene Symbol` %in% immun.mRNA)%>%

left_join(exp,by = c("ID" = "ID_REF"))%>%

select(-ID)->hp_df

hp_df[is.na(hp_df)]<-0

hp_df[is.infinite(hp_df)]<-0

colnames(celltype) <- "Immune cell type"

for (i in 1:nrow(celltype)) {

#print(i)

celltype$t.cell_type...1...[i] <- unlist(strsplit(celltype$t.cell_type...1...,split = ':')[[i]][2])

}

hp_df[rowSums(hp_df[,-1])>0,]->hp_df

hp_df_uniq <- data.frame()

for (gene in unique(hp_df$`Gene Symbol`)) {

hp_df%>%

filter(`Gene Symbol`==gene)->tmp

hp_df_uniq%>%

rbind(tmp[1,])->hp_df_uniq

}

as.data.frame(hp_df_uniq)->hp_df_uniq

rownames(hp_df_uniq) <- hp_df_uniq$`Gene Symbol`

pdf("hp.immune.mRNA.pdf",width = 14,height = 16)

pheatmap(hp_df_uniq[,-1],

annotation_col = celltype,

)

dev.off()

#miRNA

soft_mi_b%>%

filter(X8 %in% immun.miRNA)%>%

left_join(exp_mi,by = c("X1" = "ID_REF"))%>%

select(-X1)->hp_df_mi

hp_df_mi[is.na(hp_df_mi)]<-0

colnames(celltype_mi) <- "Immune cell type"

for (i in 1:nrow(celltype_mi)) {

#print(i)

celltype_mi$t.cell_type_mi...1...[i] <- unlist(strsplit(celltype_mi$t.cell_type_mi...1...,split = ':')[[i]][2])

}

hp_df_mi[rowSums(hp_df_mi[,-1])>0,]->hp_df_mi

hp_df_mi_uniq <- data.frame()

for (gene in unique(hp_df_mi$X8)) {

hp_df_mi%>%

filter(X8==gene)->tmp

hp_df_mi_uniq%>%

rbind(tmp[1,])->hp_df_mi_uniq

}

as.data.frame(hp_df_mi_uniq)->hp_df_mi_uniq

rownames(hp_df_mi_uniq) <- hp_df_mi_uniq$X8

pdf("hp.immune.miRNA.pdf",width = 10,height = 5)

pheatmap(hp_df_mi_uniq[,-1],

annotation_col = celltype_mi,

)

dev.off()

#05-SigRNA-exp-clusters

library(tidyverse)

library(pheatmap)

library(ggsignif)

setwd("analysis/04.TILsig-ceRNA")

#signature:immun.mRNA, immun.miRNA, immun.linc

immun.mRNA<-read.table("mRNA.signature",sep = ',')$immun.mRNA

immun.miRNA<-read.table("miRNA.signature",sep = ',')$immun.miRNA

immun.linc<-read.table("lncRNA.signature",sep = ',')$immun.linc

sig_mirna <- list()

for (mi in immun.miRNA) {

sig_mirna <-c(unlist(strsplit(mi,split = '//')),sig_mirna)

}

sig_mirna <- unlist(sig_mirna)

sig_mrna <- list()

for (mg in immun.mRNA) {

sig_mrna <-c(unlist(strsplit(mg,split = '//')[[1]][1]),sig_mrna)

}

sig_mrna <- unlist(sig_mrna)

sig_lncrna <- immun.linc$lncRNAs

#cluster

setwd("analysis/02.cluster_by_mutation")

clusters <- read.table("luad/m3.clusters.csv",sep = ',')

clusters$submitter_id <- substr(rownames(clusters),1,12)

clusters$clusters<- as.character(clusters$clusters)

clusters[order(clusters$clusters),]->clusters

#data

setwd("analysis/01.data")

load("mirna_lusc.rda")

miRNA_exp <- data

mRNA_exp <- load("fpkm_lusc.rda")

mRNA_exp <- data

load("mirna_luad.rda")

miRNA_exp <- data

mRNA_exp <- load("fpkm_luad.rda")

mRNA_exp <- data

#miRNA expr

setwd("analysis/05.SigceRNA-exp-cluster")

mirna_exp_clu <- data.frame(miRNA_ID = miRNA_exp$miRNA_ID)

for (i in 1:nrow(clusters)) {

cll <- grepl(paste0("reads_per_million_miRNA_mapped_",clusters[i,]$submitter_id),colnames(miRNA_exp))

df_tmp <-data.frame(C1 = miRNA_exp[,colnames(miRNA_exp)[cll]])

colnames(df_tmp) <-colnames(miRNA_exp)[cll]

mirna_exp_clu%>%

cbind(df_tmp)->mirna_exp_clu

}

rownames(miRNA_exp) <- miRNA_exp$miRNA_ID

intersect(miRNA_exp$miRNA_ID,sig_mirna)->sig_mirna_1

miRNA_exp[sig_mirna_1,colnames(mirna_exp_clu)] -> sig_mirna_exp

data.frame(sample=colnames(mirna_exp_clu),

submitter_id = substr(colnames(mirna_exp_clu),32,43))%>%

left_join(clusters)->colData_mirna

rownames(colData_mirna)<- colData_mirna$sample

colData_mirna%>%

dplyr::select(clusters)->colData_mirna

colData_mirna%>%

filter(!is.na(clusters)) -> colData_mirna

library(pheatmap)

pdf("hp.luad.miRNA.signature.TCGA.pdf",width = 8,height = 8)

pheatmap(sig_mirna_exp[,-1],

annotation_col = colData_mirna,

cluster_cols = F)

dev.off()

for (i in 1:nrow(sig_mirna_exp)) {

data.frame(t(sig_mirna_exp[i,])[-1,])->exp_tmp

exp_tmp$sample <- rownames(exp_tmp)

exp_tmp%>%

left_join(colData_mirna)->exp_tmp1

exp_tmp1$expression <- as.numeric(exp_tmp1$t.sig_mirna_exp.i......1...)

ggplot(exp_tmp1%>%

dplyr::select(-sample),aes(x=clusters,y=expression))+

ylab(label = rownames(sig_mirna_exp)[i])+

geom_boxplot(fill = color_cus[i+150])+

geom_signif(comparisons = com_luad,

map_signif_level=T,

test=wilcox.test,step_increase=0.2)+

theme_bw()->p

ggsave(filename = paste0("luad_miRNA/",rownames(sig_mirna_exp)[i],".pdf"),

width = 7,height = 3)

}

colData_mirna$sample <- rownames(colData_mirna)

color_cus <- colors()

dev.off()

#mRNA

#immunecellAI

clusters%>%

filter(clusters=="1")->cdf

sample_list = list()

for(sample in rownames(clusters)){

bc = substr(sample,1,16)

sample_add <- colnames(mRNA_exp)[grepl(bc,colnames(mRNA_exp))]

sample_list <- unlist(c(sample_list,sample_add))

}

mRNA_exp[,sample_list]->cluster1_exp

c1_exp_fpkm <- cluster1_exp@assays@data$`HTSeq - FPKM-UQ`

rownames(c1_exp_fpkm) <-rownames(cluster1_exp)

colnames(c1_exp_fpkm) <- colnames(cluster1_exp)

write.table(c1_exp_fpkm,

file = "cluster1.lusc.exp.txt",

sep = '\t')

genelist <- mRNA_exp@rowRanges$ensembl_gene_id[mRNA_exp@rowRanges$external_gene_name %in% sig_mrna]

mRNA_exp[genelist,sample_list] ->sig_mrna_exp

sig_mrna_exp_fpkm <- sig_mrna_exp@assays@data$`HTSeq - FPKM-UQ`

rownames(sig_mrna_exp_fpkm) <-rownames(sig_mrna_exp)

colnames(sig_mrna_exp_fpkm) <- colnames(sig_mrna_exp)

data.frame(sample = sample_list,submitter_id = substr(sample_list,1,12))%>%

left_join(clusters)%>%

dplyr::select(clusters)->colData_mrna

fpkmToTpm <- function(fpkm)

{

exp(log(fpkm) - log(sum(fpkm)) + log(1e6))

}

sig_mrna_exp_tpm <- as.data.frame (apply(sig_mrna_exp_fpkm, 2, fpkmToTpm))

rownames(colData_mrna) <- sample_list

pdf(file = "hp.luad.mRNA.signature.TCGA.pdf",width = 8,height = 8)

pheatmap(sig_mrna_exp_tpm,annotation_col = colData_mrna,cluster_cols = F)

dev.off()

colData_mrna$sample <- rownames(colData_mrna)

com_lusc <- list(c("1", "3"),

c("2","13"),

c("3","16"),

c("9","13"),

c("15","13"))

com_luad <- list(c("1","9"),c("5","12"),c("6","7"),c("7","9"),c("8","12"),

c("9","12"),c("12","14"),c("12","17"))

for (i in 1:nrow(sig_mrna_exp_tpm)) {

genename <- mRNA_exp@rowRanges$external_gene_name[mRNA_exp@rowRanges$ensembl_gene_id==rownames(sig_mrna_exp_tpm)[i]]

data.frame(t(sig_mrna_exp_tpm[i,])[-1,])->exp_tmp

exp_tmp$sample <- rownames(exp_tmp)

exp_tmp%>%

left_join(colData_mrna)->exp_tmp1

exp_tmp1$expression <- as.numeric(exp_tmp1$t.sig_mrna_exp_tpm.i......1...)

ggplot(exp_tmp1%>%

dplyr::select(-sample),aes(x=clusters,y=expression))+

ylab(label = genename)+

geom_boxplot(fill = color_cus[i+105])+

geom_signif(comparisons = com_luad,

map_signif_level=T,

test=wilcox.test,step_increase=0.2)+

theme_bw()->p

ggsave(filename = paste0("luad_mRNA/",genename,".pdf"),

width = 7,height = 3)

}

#lncRNA

lncrna_genelist1 <- mRNA_exp@rowRanges$ensembl_gene_id[mRNA_exp@rowRanges$external_gene_name %in% sig_lncrna]

lncrna_genelist2 <- intersect(mRNA_exp@rowRanges$ensembl_gene_id,sig_lncrna)

lncrna_exp <- mRNA_exp[c(lncrna_genelist1,lncrna_genelist2),sample_list]

lncrna_exp_fpkm <- lncrna_exp@assays@data$`HTSeq - FPKM-UQ`

rownames(lncrna_exp_fpkm) <- rownames(lncrna_exp)

colnames(lncrna_exp_fpkm) <- colnames(lncrna_exp)

data.frame(sample = sample_list,submitter_id = substr(sample_list,1,12))%>%

left_join(clusters)%>%

dplyr::select(clusters)->colData_lncrna

rownames(colData_lncrna) <- sample_list

lncrna_exp_tpm <- as.data.frame (apply(lncrna_exp_fpkm, 2, fpkmToTpm))

dim(lncrna_exp_tpm)

pdf(file = "hp.luad.lncRNA.signature.col.TCGA.pdf",width = 8,height = 8)

pheatmap(lncrna_exp_tpm,annotation_col = colData_lncrna,cluster_cols = T)

dev.off()

colData_lncrna$sample <- rownames(colData_lncrna)

for (i in 1:nrow(lncrna_exp_tpm)) {

genename <- mRNA_exp@rowRanges$external_gene_name[mRNA_exp@rowRanges$ensembl_gene_id==rownames(sig_mrna_exp_tpm)[i]]

data.frame(t(lncrna_exp_tpm[i,])[-1,])->exp_tmp

exp_tmp$sample <- rownames(exp_tmp)

exp_tmp%>%

left_join(colData_mrna)->exp_tmp1

exp_tmp1$expression <- as.numeric(exp_tmp1$t.lncrna_exp_tpm.i......1...)

ggplot(exp_tmp1%>%

dplyr::select(-sample),aes(x=clusters,y=expression))+

ylab(label = genename)+

geom_boxplot(fill = color_cus[i+105])+

geom_signif(comparisons = com_luad,

map_signif_level=T,

test=wilcox.test,step_increase=0.2)+

theme_bw()->p

ggsave(filename = paste0("luad_lncRNA/",genename,".pdf"),

width = 7,height = 3)

}

#06-Immune-features

library(reshape2)

library(ggplot2)

setwd("analysis/06.cluster-immunescore")

#immunecellAI

lusc_immai <- readr::read_tsv("immunecellai/LUSC_self.txt",col_names = T,)

lusc_immai$sample <- gsub("\\.","-",lusc_immai$sample)

luad_immai <- readr::read_tsv("immunecellai/LUAD_self.txt",col_names = T)

luad_immai$sample <- gsub("\\.","-",luad_immai$sample)

setwd("analysis/02.cluster_by_mutation")

clusters <- read.table("luad/m3.clusters.csv",sep = ',')

clusters$ID <- substr(rownames(clusters),1,15)

clusters$clusters <- as.character(clusters$clusters)

clusters$sample_s <- clusters$ID

lusc_immai$sample_s <- substr(lusc_immai$sample,1,15)

luad_immai$sample_s <- substr(luad_immai$sample,1,15)

clusters%>%

left_join(luad_immai,by = c("sample_s" = "sample_s"))%>%

dplyr::select(-sample,-sample_s,-ID)->immai_df

rownames(immai_df) <- rownames(clusters)

dim(immai_df)

dim(clusters)

for(i in 2:length(immai_df)){

cell_type = colnames(immai_df)[i]

df<-immai_df[,c(1,i)]

colnames(df) <- c("clusters","celltype")

ggplot(df,aes(x=clusters,y=celltype))+ylab(label = cell_type)+

geom_boxplot(fill = color_cus[i+10])+theme_bw()->p

ggsave(filename = paste0("immunecellai/dist.luad.",cell_type,".pdf"),p,

width = 8,height = 1.2)

}

color_cus <- colors()

#estimate score

lusc_ssis <- readr::read_tsv("estimate_score/lung_squamous_cell_carcinoma_RNAseqV2.txt",col_names = T)

luad_ssis <- readr::read_tsv("estimate_score/lung_adenocarcinoma_RNAseqV2.txt",col_names = T)

clusters$ID <- substr(rownames(clusters),1,15)

clusters$clusters <- as.character(clusters$clusters)

clusters%>%

left_join(luad_ssis)%>%

dplyr::select(-sample_s,-ID)->ssis_df

rownames(ssis_df) <- rownames(clusters)

melt(ssis_df)->ssis_df1

ggplot(ssis_df1,aes(x=clusters,y=value,fill=variable))+

geom_boxplot()+

theme_bw()->p

ggsave(filename = "estimate_score/dist.luad.pdf",p,width = 11,height = 5)

#07-survival

#LUSC

clin_lusc, dataSmNT,dataSmTP

expr,mirna_exp_clu

head(mirna_exp_clu)

rownames(mirna_exp_clu) <- mirna_exp_clu$miRNA_ID

mirna_exp_clu%>%

dplyr::select(-miRNA_ID)->mirna_exp_clu_surdf

colnames(mirna_exp_clu_surdf) <- substr(colnames(mirna_exp_clu_surdf),32,46)

expr <- mirna_exp_clu_surdf

intersect(rownames(expr),gene_symbol)

#mutation sample

maf_luad%>%

filter(Hugo_Symbol %in% c("ATM","TP53"))%>%

dplyr::select(Tumor_Sample_Barcode)->mut_sample_df

mut_sample<- intersect(substr(unique(mut_sample_df$Tumor_Sample_Barcode),1,15),colnames(expr))

sur<-function(gene_name,gene_symbol){

gene_symbol <-"hsa-mir-185"

#outdir <- c("C:/Users/FuXin/Desktop/FU/PROJECT/Prostate_Cancer/6.survival/Survival_Month/chemokine")

outdir <- paste0("C:/Users/FuXin/OneDrive/FU/LiKe/LungCancer-202112-202206/analysis/08.survival")

setwd(outdir)

gene_exp_TP_marker<-expr[gene_symbol,intersect(substr(dataSmTP,1,15),mut_sample)]

#gene_exp_TP_marker<-expr[gene_symbol,intersect(substr(dataSmTP,1,15),colnames(expr))]

names(gene_exp_TP_marker) <- sapply(strsplit(names(gene_exp_TP_marker),'-'),function(x) paste0(x[1:3],collapse="-"))

gene_exp_TP_marker_t <- t(gene_exp_TP_marker)

clin_luad$marker <- gene_exp_TP_marker_t[match(clin_luad$submitter_id,rownames(gene_exp_TP_marker_t)),]

clin_luad%>%

filter(!is.na(marker))->clin_luad.filt

df<-subset(clin_luad.filt,

select =c(submitter_id,vital_status,days_to_death,days_to_last_follow_up,marker))

df$days_to_death[is.na(df$days_to_death)] <- 0

df$days_to_last_follow_up[is.na(df$days_to_last_follow_up)] <- 0

df$days=as.numeric(df$days_to_death)+as.numeric(df$days_to_last_follow_up)

df$days_to_death=round(df$days/30,2)

df$exp <- ''

if(median(df$marker)==0){

print("Undo")

}else{

df[df$marker >= median(df$marker),]$exp <- "H"

df[df$marker < median(df$marker),]$exp <- "L"

TCGAanalyze_survival(df,

clusterCol="exp",

legend = gene_symbol,

risk.table = FALSE,

conf.int = FALSE,

color = c("Dark2"),

#main = gene_symbol,

xlab = "Time since diagnosis (Monthes)",

dpi=1200,

height = 4, width = 5,

#dpi = 300,

filename = paste0(gene_symbol,"_survival.mut.pdf"))

}

}
